# *SCI1* Is a Direct Target of AGAMOUS and WUSCHEL and Is Specifically Expressed in the Floral Meristematic Cells

**DOI:** 10.3389/fpls.2021.642879

**Published:** 2021-03-18

**Authors:** Joelma O. Cruz, Juca A. B. San Martin, Greice Lubini, Edward J. Strini, Rómulo Sobral, Vitor F. Pinoti, Pedro B. Ferreira, Vanessa Thomé, Andréa C. Quiapim, Marcelo C. Dornelas, Maria Cristina S. Pranchevicius, Francisco Madueño, M. Manuela R. Costa, Maria Helena S. Goldman

**Affiliations:** ^1^Departamento de Biologia, Faculdade de Filosofia, Ciências e Letras de Ribeirão Preto, Universidade de São Paulo, Ribeirão Preto, Brazil; ^2^PPG-Genética, Faculdade de Medicina de Ribeirão Preto, Universidade de São Paulo, Ribeirão Preto, Brazil; ^3^Biosystems and Integrative Sciences Institute, Plant Functional Biology Center, University of Minho, Braga, Portugal; ^4^Departamento de Biologia Vegetal, Instituto de Biologia, Universidade de Campinas, Campinas, Brazil; ^5^Departamento de Genética e Evolução, Universidade Federal de São Carlos, São Carlos, Brazil; ^6^Instituto de Biología Molecular y Celular de Plantas, CSIC-UPV, Valencia, Spain

**Keywords:** co-expression, floral determinacy, flower development, meristematic cells, *Nicotiana tabacum*, transcriptional control

## Abstract

The specified floral meristem will develop a pre-established number of floral organs and, thus, terminate the floral meristematic cells. The floral meristematic pool of cells is controlled, among some others, by WUSCHEL (WUS) and AGAMOUS (AG) transcription factors (TFs). Here, we demonstrate that the *SCI1* (*Stigma/style cell-cycle inhibitor 1*) gene, a cell proliferation regulator, starts to be expressed since the floral meristem specification of *Nicotiana tabacum* and is expressed in all floral meristematic cells. Its expression is higher in the floral meristem and the organs being specified, and then it decreases from outside to inside whorls when the organs are differentiating. *SCI1* is co-expressed with *N. tabacum WUSCHEL* (*NtWUS*) in the floral meristem and the whorl primordia at very early developmental stages. Later in development, *SCI1* is co-expressed with *NAG1* (*N. tabacum AG*) in the floral meristem and specialized tissues of the pistil. *In silico* analyses identified *cis*-regulatory elements for these TFs in the *SCI1* genomic sequence. Yeast one-hybrid and electrophoresis mobility shift assay demonstrated that both TFs interact with the *SCI1* promoter sequence. Additionally, the luciferase activity assay showed that NAG1 clearly activates *SCI1* expression, while NtWUS could not do so. Taken together, our results suggest that during floral development, the spatiotemporal regulation of *SCI1* by NtWUS and NAG1 may result in the maintenance or termination of proliferative cells in the floral meristem, respectively.

## Introduction

The maintenance and termination of the floral meristem are orchestrated by a complex network of elements that involve transcription factors (TFs), hormonal signaling, and cell cycle control genes (Jha et al., 2020). In the Arabidopsis floral meristem, the undetermined proliferation of cells is dependent on the expression level of *WUSCHEL* (*WUS*), a homeobox TF gene expressed in the organizing center (OC) (Laux et al., 1996). The OC is composed of pluripotent cells maintained until the specification of the four floral whorls: sepals, petals, stamens, and carpels (Sharma et al., 2003). During the early stages of floral development, the feedback between *WUS-CLAVATA* (*CLV*) sustains the homeostasis of the floral meristem (Zhou et al., 2015). *WUS* is transcribed in the OC, and the protein migrates to the outer layers of the floral meristem, where it activates *CLV3* (Yadav et al., 2013). *WUS* blocks cell differentiation by inactivating genes, such as the *ARR7/ARR15* (*ARABIDOPSIS RESPONSE REGULATOR*) that are mediators of the auxin control of cytokinin signaling (Leibfried et al., 2005; Zhao et al., 2010). Another example is the role of WUS in inhibiting genes in the auxin biosynthesis pathway (Mano and Nemoto, 2012), preventing cell differentiation (Yadav et al., 2013). The interaction between these pathways guarantees the balance between proliferation and differentiation, which is essential for the correct formation of the flower (Sun et al., 2009).

In the floral meristem, pluripotent cells do not multiply indefinitely as in the shoot apical meristem. The expression of *WUS* is down regulated during the specification of the fourth whorl in the center of the floral meristem (Mayer et al., 1998). The specification of carpels is established by AGAMOUS (AG), a MADS-box TF (Bowman et al., 1989; Ó’Maoiléidigh et al., 2014). *AG* is also responsible for terminating the proliferation of undetermined cells in the center of the floral meristem (Lenhard et al., 2001). *AG* expression is activated due to the cooperation between LEAFY (LFY) and WUS TFs that bind to the second *AG* intron (Lohmann et al., 2001). Once activated, AG suppresses *WUS* expression by recruiting the CURLY LEAF protein (CLF) that is part of the polycomb repressive complex 2 (PRC2) and adds a tri-methylation to lysine 27 in histone 3 (H3K27me3), thus inactivating *WUS* expression (Liu et al., 2011). AG also activates the expression of *KNUCKLES* (*KNU*), which encodes a C2H2-zinc finger TF that directly represses *WUS* expression at stage 6 of *Arabidopsis thaliana* flower development (Sun et al., 2009), putting an end to undifferentiated proliferation. Although widely studied, there are still gaps in the knowledge involving the signaling processes downstream of *WUS* and *AG* that ultimately result in the termination of the pool of meristematic cells and differentiation.

*Stigma/style cell-cycle inhibitor 1* (*SCI1*) was previously described as a gene preferentially expressed in stigma/style that controls cell proliferation in the upper pistil of *N. tabacum* and *A. thaliana* (DePaoli et al., 2011, 2014). *N. tabacum SCI1^*Ri*^* silencing plants and *A. thaliana sci1* mutants presented elongated styles and increased stigmatic areas. These phenotypes resulted from increased cell number (DePaoli et al., 2011, 2014). Furthermore, *SCI1*^*Ri*^ plants have accelerated cell differentiation in the stigma surface, consistent with a role for SCI1 in triggering differentiation through cell proliferation control (DePaoli et al., 2011). There is a relationship between *SCI1* and the auxin signaling pathway not yet understood. The *NtAux/IAA19*, *NtAux/IAA13*, and *NtARF8* expression levels were affected by *SCI1* expression, especially in plants overexpressing *SCI1* (DePaoli et al., 2012). In *A. thaliana*, the altered phenotypes of *sci1*, *yuc2yuc6*, and *npy1* mutants cannot be distinguished. Additionally, overexpression of *SCI1* in a *yuc2yuc6sci1* background restores the wild-type phenotype. These findings point to an overlap of *SCI1* and *YUCCA* genes in the auxin signaling pathway during pistil development in Arabidopsis (DePaoli et al., 2014).

Due to its key role in cell proliferation regulation and early expression in pistil development, we asked when *SCI1* starts to be expressed during flower development. In this study, we carried out detailed analyses of *SCI1* expression since flower meristem specification, by *in situ* hybridization detection of the endogenous transcript, and the expression driven by *SCI1* genomic sequence translationally fused to GFP in transgenic plants. *In silico* analyses of the *SCI1* genomic sequence indicated the presence of AG and WUS cis-regulatory elements. We demonstrated the co-expression of *SCI1* and *N. tabacum WUSCHEL* (*NtWUS*), as well as *SCI1* and *N. tabacum AG* (*NAG1*), the *N. tabacum* orthologs of WUS (Li et al., 2018; Zhou et al., 2018) and AG (Kempin et al., 1993), respectively, in the flower meristem. The binding of these two TFs to the *SCI1* promoter sequence was confirmed by yeast one-hybrid (Y1H) and electrophoresis mobility shift assay (EMSA), and *in planta* luciferase activity assay for NAG1. We gathered evidence showing that *SCI1* expression is activated by the TF NAG1. As *SCI1* is a regulator of cell proliferation/differentiation, we discuss the hypothesis of *SCI1* being an effector of the termination process of the floral meristem of *N. tabacum*, which is under the control of NAG1.

## Results

### *SCI1* Starts to Be Expressed at the Specification of the Floral Meristem and Maintains Its Expression in Proliferative Cells

**FIGURE 1:**
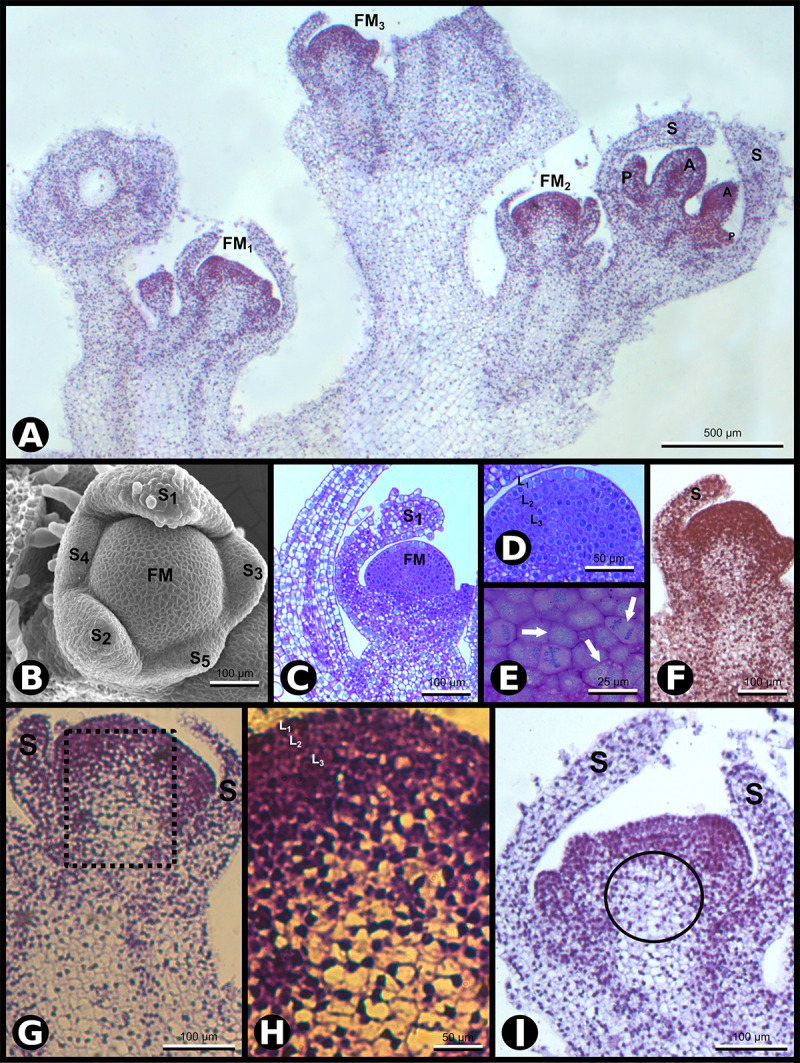
*SCI1* expression during *Nicotiana tabacum* early floral development. **(A)** *In situ* hybridization of inflorescence apex with *SCI1* antisense probe. Four floral buds are observed. Scale bar: 500 μm. **(B)** Scanning electron microscopy (SEM) showing the asynchronous emergence of the sepals (S1–S5) in a flower meristem and sepal S1 with trichomes (stage –9, here defined). Scale bar: 100 μm. **(C)** Bright-field microscopy showing a longitudinal section of a very young flower bud at stage –9. Scale bar: 100 μm. **(D)** A higher magnification view of the flower bud in C, in which the three meristematic cell layers (L1, L2, and L3) are seen. Scale bar: 50 μm. **(E)** A higher magnification view of the flower meristem shown in **(D)**, in which cell divisions are visible (arrows). Scale bar: 25 μm. **(F–I)** *In situ* hybridization with *SCI1* antisense probe of very young flower buds, even before stage –7, the youngest developmental stage defined by Koltunow et al. (1990). **(F)** Flower meristem with emerging sepals (stage –10, here defined). This is a higher magnification of FM3 from **(A)**. Scale bar: 100 μm. **(G)** Flower meristem with sepal primordia (stage –9, here defined). This is a higher magnification of FM2 from **(A)**. Scale bar: 100 μm. **(H)** A higher magnification view of the marked area in **(G)**. The meristematic cell layers (L1, L2, and L3) are identifiable. Scale bar: 50 μm. **(I)** Flower meristem with emerging petals and anthers, at stage –8 (here defined). This is a higher magnification of FM1 from **(A)**. Scale bar: 100 μm. Floral meristem (FM), sepals (S). Compare the image shown in **(F)** with the images **(G–I)** and observe the reduced *SCI1* expression in the OC [encircled in **(I)**]. Negative controls of flower buds in equivalent developmental stages are shown in Supplementary Figure 1.

Our previous work demonstrated that the *SCI1* gene is highly expressed at the very early developmental stages of *N. tabacum* pistils (DePaoli et al., 2011). Therefore, we became interested in studying when *SCI1* expression starts. For this purpose, *in situ* hybridizations were performed using *SCI1* antisense transcripts as a probe in histological sections of the inflorescence apex. The results demonstrate that *SCI1* is expressed since floral meristem initiation and emergence (Figure 1A).

To have a detailed understanding of the *N. tabacum* flower meristem development and establish a parallel with *SCI1* expression, we also implemented anatomical and histological analyses in conjunction with the *in situ* hybridization experiments. At the very early stages of development [stage −9, here defined based on the earlier stage described by Koltunow et al. (1990) as stage −7], five sepal primordia arise sequentially at the edges of the floral meristem, as documented by scanning electron microscopy (SEM) (Figure 1B). The floral meristem, seen at the center, is organized in three cell layers (L1, L2, and L3), easily distinguished in anatomical sections observed by bright field microscopy (Figures 1C,D). Several cell divisions are clearly visible at the meristematic cell layers (Figure 1E). The sepal primordia arise asynchronously and clockwise in divergent angles of approximately 144 degrees relative to the previous primordium (Figure 1B). After reaching a specific size, the sepal primordia show trichomes at the abaxial side (Figures 1B,C). At this very early stage, *SCI1* is expressed at the emergence of sepal primordia (Figures 1F,G) and at high levels in all cell layers of the central floral meristem (Figures 1F–I). When the sepals are specified, *SCI1* expression decreases in the region below the third layer, the area described as the OC (Figures 1G–I).

**FIGURE 2:**
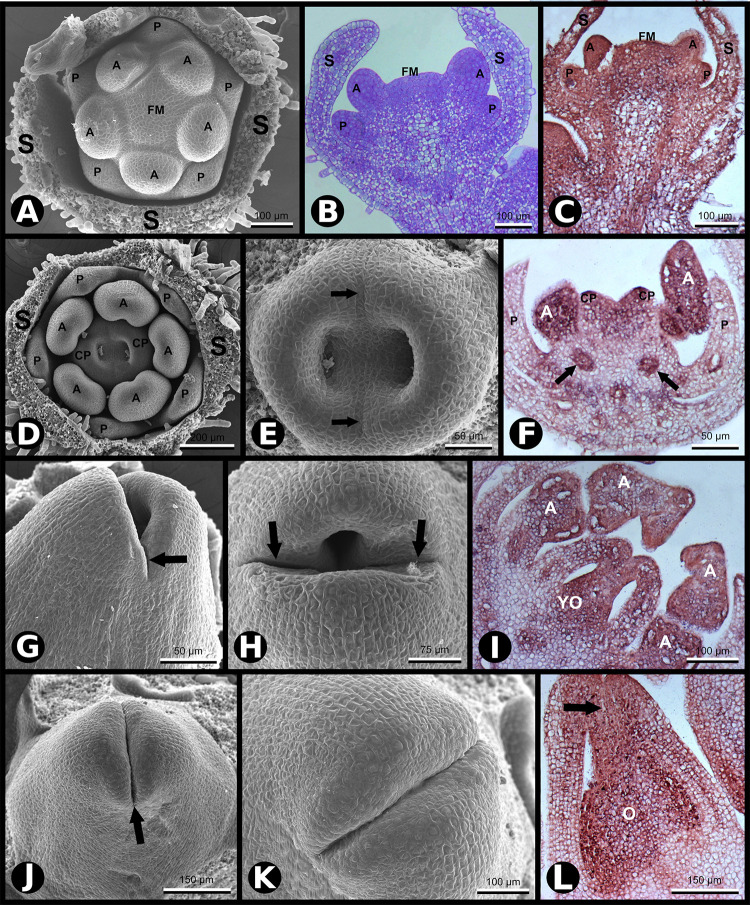
*SCI1* expression during later stages of floral development (continuation of the stages shown in Figure 1). **(A)** SEM of a flower bud in which petals and anthers are emerging (advanced stage –8). Scale bar: 100 μm. **(B)** Bright-field microscopy showing a longitudinal section of a flower bud in a developmental stage equivalent to the one shown in **(A)**. Scale bar: 100 μm. **(C)** *In situ* hybridization of a flower bud (advanced stage –8) with *SCI1* antisense probe. Scale bar: 100 μm. **(D)** SEM of a flower bud at stage –7 (as defined by Koltunow et al., 1990), in which carpels are emerging. Scale bar: 200 μm. **(E)** A higher magnification view of the flower bud shown in **(D)**, in which the fusion lines are visible (arrows). Scale bar: 50 μm. **(F)** *In situ* hybridization of a flower bud at stage –7/–6, with *SCI1* antisense probe. Arrows point to ovary locules. Scale bar: 50 μm. **(G,H)** SEM of flower buds at stage –6; carpels fused at the base and not yet fused at the top. Scale bars: 50 μm **(G)** and 75 μm **(H)**. **(I)** *In situ* hybridization of a flower bud at late stage –6, with *SCI1* antisense probe. Scale bar: 100 μm. **(J,K)** SEM of flower buds at stage –5; carpels already fused at the top; the fusion region is a site of intense cell proliferation. Scale bars: 150 μm **(J)** and 100 μm **(K)**. **(L)** *In situ* hybridization with *SCI1* antisense probe of a flower bud at late stage –5; style beginning to form (arrow). Scale bar: 150 μm. Floral meristem (FM), sepals (S), petals (P), anther (A), carpels (C), carpel primordia (CP), ovary (O), young ovary (YO). Negative controls of flower buds in equivalent developmental stages are shown in Supplementary Figure 1.

At developmental stage −8 (here defined), petal and stamen primordia upraise synchronously and almost simultaneously from the floral meristem, while sepals grow toward each other. At this stage, cells of the petal primordia, stamen primordia, and the central floral meristem have characteristics of meristematic cells (Chang and Sun, 2020), in contrast to the sepal cells that are differentiating (Figure 2A). At this stage, the floral meristem is restricted to the area in which the carpel primordia will form (Figure 2B). The five-petal primordia emerge in alternate positions in relation to the sepal primordia. Similarly, the five stamen primordia arise in alternate places with regard to the petal primordia. Therefore, the stamen primordia are positioned in the same direction as the sepal primordia. Despite the almost simultaneous upraise of petal and stamen primordia, the latter seem to grow faster than the petals at this stage. During flower development, *SCI1* expression is temporally and spatially regulated (Figures 1, 2). At advanced stage −8, *SCI1* is highly expressed in the petal and stamen primordia, as well as in the remaining central floral meristem (Figure 2C). Still, its expression has already decreased at the differentiating sepals.

Later in development (stage −7), the two carpel primordia emerge, and their invaginations are clearly seen where the two ovary locules will develop (Figures 2D,E). In *N. tabacum*, the carpels fuse postgenitally, and it is possible to observe the carpel primordia growing toward each other to converge at the top, while the base is already connected (Figure 2E). At an equivalent stage, Chang and Sun (2020) identified cell divisions in the L1 layer and deeper layers of the *N. tabacum* carpels. At stage −7, the *SCI1* signal is much weaker in sepals and petals that are differentiating (Figure 2F). Meanwhile, specified anthers are developing, and *SCI1* is strongly detected in this whorl (Figure 2F). In the carpel primordia, the *SCI1* signal is intense, especially in the upper part where fusion occurs. Within the carpels, *SCI1* is also expressed in the ovary locules (Figure 2F), a region with meristematic cells that will develop into ovules, suggesting a function for *SCI1* in ovule development.

Development progresses (stage −6), and carpel primordia are growing toward each other (Figures 2G,H) and will fuse, where cells will continue to divide to give rise to the style and stigma (Figures 2J,K). At this developmental stage, *SCI1* expression is obvious in anthers and carpels (Figure 2I), while already very weak at sepals and petals.

At stage −5, carpels are already fused at the top of the ovary (Figures 2J–L). The fused carpels show high *SCI1* expression (Figure 2L), which is clearly visible on the top, where cell divisions will give rise to the style and stigma. The presence of *SCI1* transcripts is evident in the inner part of the developing style, which will become the stylar transmitting tissue (STT), and is also very clear in the ovary locules (Figure 2L).

As development progresses (stage −2), it is possible to observe intense cell proliferation along the carpel fusion line (Supplementary Figure 2). The stigma lobules are already established, the upper surface cells start differentiating as stigmatic papilla while, internally, the stigmatic secretory zone (SSZ) and STT are differentiating along the fusion line (Supplementary Figure 2). The intense cell proliferation continues along the fusion line, resembling a “volcano eruption,” resulting in the folding of the stigma to the “umbrella-like” structure, typical of the *N. tabacum* flower. These inner proliferating tissues (SSZ and STT) are the sites of *SCI1* expression, as we showed previously (DePaoli et al., 2011).

According to our results, *SCI1* is expressed since flower meristem specification and at all floral whorl primordia; its expression is restricted to cells with proliferative capacity and decreases during later developmental stages toward differentiation.

### The Genomic Sequence of *SCI1* Drives Expression Specifically to the Floral Meristem and Its Proliferative Cells

**FIGURE 3:**
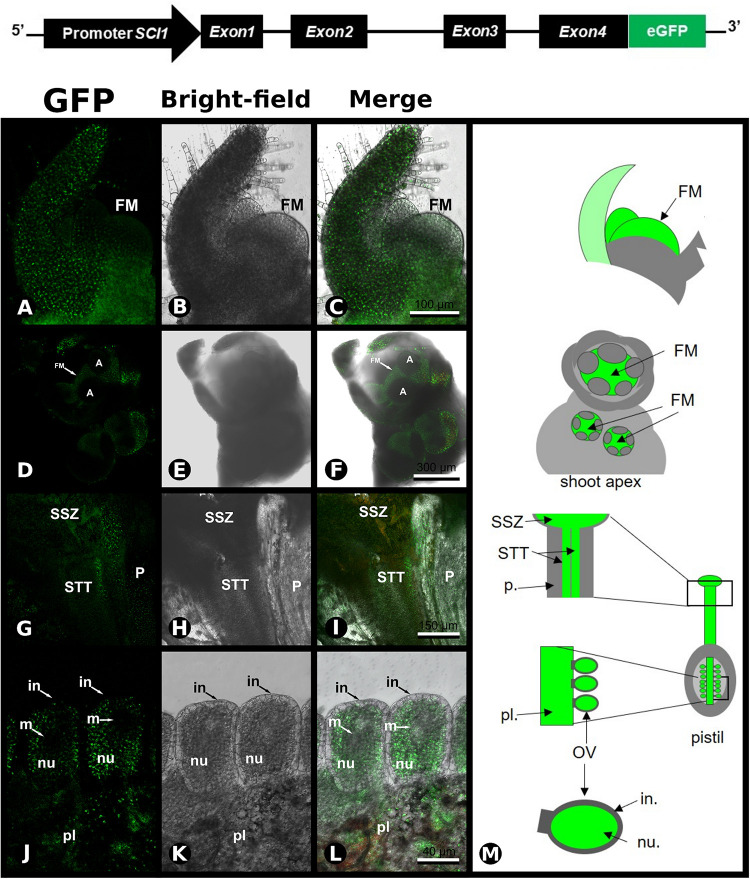
Expression of SCI1-GFP in transgenic plants. Upper panel – Schematic representation of the construct used to produce SCI1-GFP transgenic plants. **(A–C)** Confocal microscopy of a crushed flower meristem (transgenic plant SCI1gen-GFP17.1). In the first focal plane, a sepal is seen, and behind, the floral meristem. Scale bar: 100 μm. **(D–F)** Inflorescence apex containing three floral buds; observe the intense GFP fluorescence at the floral meristem (FM). Scale bar: 300 μm. **(G–I)** Stigma/style of transgenic plant SCI1gen-GFP108.3, basolateral longitudinal section of the upper part of the pistil; observe intense GFP fluorescence at the stylar transmitting tissue (STT), and the stigmatic secretory zone (SSZ); very weak GFP fluorescence at the parenchyma tissue. Scale bar: 150 μm. **(J–L)** Ovary section of transgenic plant SCI1gen-GFP5.1; observe the intense GFP fluorescence at the placenta (pl) and nucellus (nu); no GFP was detected at the megaspore region (m) and integument (in). Scale bar: 40 μm. **(M)** Schematic representations of the flower regions analyzed by confocal microscopy. Floral meristem (FM), parenchyma (p.), stigmatic secretory zone (SSZ), stylar transmitting tissue (STT), ovule (OV).

To study the transcriptional control of *SCI1* expression, we have produced 17 independent transgenic plants containing the genomic sequence of *SCI1* (∼4.5 kb) in translational fusion with GFP (Figure 3). The *SCI1* genomic sequence contains a 1.9 kb sequence upstream of the initial ATG codon (here designated as *SCI1* promoter), the four exons, and three introns of the *N. tabacum* gene, from which the stop codon was removed for the translational GFP fusion. In all transgenic plants containing *SCI1prom:SCI1-GFP*, it was possible to detect SCI1-GFP at the floral meristems by confocal fluorescence microscopy. No GFP fluorescence was detected at the shoot apical meristem and root meristem, as well as in leaves, stems, and roots of mature transgenic plants. As seen using *in situ* hybridization for *SCI1* endogenous expression, SCI1-GFP was detected in all floral meristem cells (Figures 3A–F). Figures 3D–F show an inflorescence apex, with two young floral meristems and one floral bud at a later developmental stage. It is visible that the GFP fluorescence is limited to the cells of the young floral meristems and to the most recent (younger) developing primordia of the floral bud (Figures 3D–F), while its detection is already decreased in the more developed and differentiated external organs (Figures 3D–F). Therefore, during floral development, *SCI1* is always expressed at higher levels at the central portion of the flower, with decreasing levels in the outer whorls as they develop. At stage −3, SCI1-GFP is detected at petals, anthers, and pistil (Supplementary Figure 3A). At stage −2, SCI1-GFP is observed in the same whorls, but fluorescence is considerably reduced in petals (Supplementary Figure 3B).

In the carpels, SCI1-GFP was detected in the STT, in the SSZ, and, in much lower intensity/quantity, in the parenchyma tissue of the style (Figures 3G–I). Within the ovaries, SCI1-GFP was observed in the placenta and nucellus, while no GFP was noticed in the ovule integument and megaspore region, or very weakly detectable (Figures 3J–L). The results obtained with the SCI1-GFP protein in transgenic plants reproduce the endogenous *SCI1* mRNA localization, as demonstrated by *in situ* hybridizations in wild-type plants. Therefore, we conclude that the *SCI1* genomic sequence contains the necessary and sufficient *cis*-acting elements for the proper transcriptional regulation of *SCI1* expression. Taken together, our results demonstrate that *SCI1* is exclusively expressed at the floral meristem and in the proliferative cells of the floral organ primordia.

### *Cis*-Acting Elements Identified in the *SCI1* Genomic Sequence

The *SCI1* genomic sequence considered in this work comprises 4455 bp, including 1937 bp upstream of the initial ATG codon (here denominated as *SCI1* promoter), four exons, and three introns. The nucleotide A of the start codon was considered a +1 position. This genomic sequence was analyzed in the PlantRegMap software^1^ using the *N. tabacum* database. In the analysis using a *p*-value ≤ 1e-5 threshold, putative *cis*-acting regulatory elements were identified for binding of different TFs belonging to several families (for details, see Supplementary Table 1). Among the putative TFs to regulate *SCI1* expression with *cis*-elements upstream of the initial ATG were: APETALA1 (AP1), which contributes to the establishment of the floral meristem; SEPALATTA3 (SEP3), involved in the specification of floral whorls; WUS and AINTEGUMENTA-like 6 (AIL-6), related to the control of cell proliferation and differentiation. Downstream of the initial ATG, sites for the following putative TFs were found: E2F/DP, related to the cell cycle; LATERAL ORGAN BOUNDARIES (LOB), with an essential role in plant growth and development; SUPPRESSOR OF OVEREXPRESSION OF CO1 (SOC1)-like, a central regulator of flowering time; AINTEGUMENTA-like AIL1, that binds to the promoter of key cell cycle genes; JACKDAW and MAGPIE, which are involved in the regulation of tissue boundaries and asymmetric cell division (Welch et al., 2007); LFY, a master regulator of flowering; and the AUXIN RESPONSE FACTOR 5 (ARF5). Interestingly, in two different positions of the *SCI1* genomic sequence, there is an overlap between AIL1 and SOC1 putative binding sites.

Moreover, considering a *p*-value ≤ 1e-3 threshold, we found three putative binding sites for AG in the *SCI1* promoter region. Two additional putative binding sites for WUS were identified, one at *SCI1* promoter, around 200 bp upstream the initial ATG, and another on the third intron. The regulatory elements identified in the *SCI1* genomic sequence point to involvement in the cell proliferation regulatory pathway, as well as in the regulation of flowering and flower development.

### *SCI1* Is Co-expressed With *AGAMOUS* (*NAG1*) e *NtWUS* in the Floral Meristematic Cells

**FIGURE 4:**
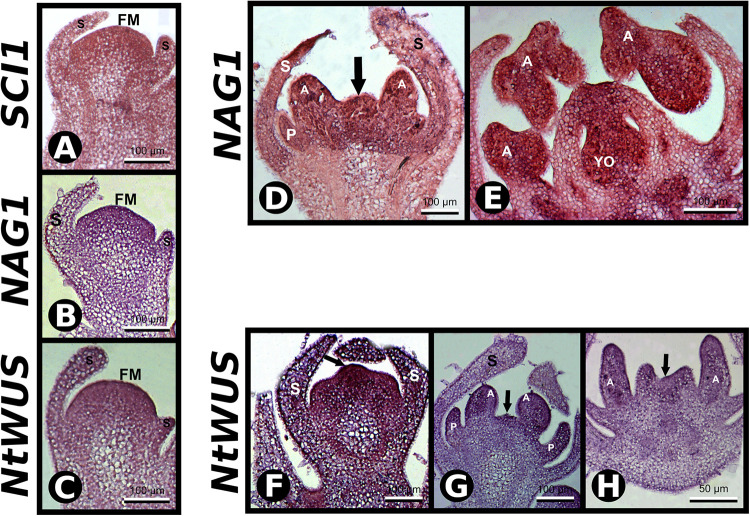
*In situ* hybridization with *SCI1*, *NAG1*, and *NtWUS* antisense probes. **(A–C)** *In situ* hybridization in very young floral buds with the indicated probes. Scale bars: 100 μm. **(D,E)** *In situ* hybridization in floral buds with *NAG1* antisense probe. Stages –8 and –5 of flower development, respectively. Scale bars: 100 μm. **(F–H)** *In situ* hybridization with *NtWUS* antisense probe. **(F,G)** Flower buds younger than stage –7. Scale bars: 100 μm. **(H)** Stage –7 of flower development. Scale bars: 50 μm. Floral meristem (FM), sepals (S), petals (P), anther (A), young ovary (YO). Negative controls of flower buds in equivalent developmental stages are shown in Supplementary Figure 1.

As putative *cis*-acting elements for AG and WUS binding were found in the *SCI1* genomic sequence, and the *SCI1* expression pattern is similar to both of these genes (Kempin et al., 1993; Zhou et al., 2018), we performed *in situ* hybridization experiments to detect their transcripts in histological sections of the same flowers used for *SCII* probes (Figure 4). At stage −8, *NAG1* transcripts were observed in stamen primordia and the center of the floral meristem (Figure 4D). At a later developmental stage, in which the carpelar leaves are fused (stage −5), *NAG1* is expressed in the ovary and in the cells that will give rise to the style (Figure 4E), as previously described (Kempin et al., 1993). The same expression pattern was observed for *SCI1* transcripts (Figure 2I). *NtWUS* is expressed at the full extension of the floral meristem when only the sepal primordia are observed (Figure 4F). At a later developmental stage, *NtWUS* transcripts were detected in the primordia of petals and anthers, as well as in the remaining floral meristem (Figure 4G). At stage −7, *NtWUS* is weakly expressed in the stamen and carpel primordia (Figure 4H). Our results for *NtWUS* expression corroborate those previously described by Zhou et al. (2018). The co-expression of these genes is clear when equivalent developmental stages are compared. *NtWUS*, *SCI1*, and *NAG1* are co-expressed mainly at the central floral meristem and primordia of the floral organs. At the same time, the co-expression of *SCI1* and *NAG1* is more evident at the floral meristem, carpelar leaves, and ovary locules. The co-expression of *SCI1* with *NtWUS* and *NAG1*, essential for floral meristem maintenance, reproductive organs specification, and floral meristem termination, respectively, points toward interconnection of these genes in the cell proliferation control at the floral meristem.

### *SCI1* Is a Direct Target of NAG1 and NtWUS Transcription Factors

**FIGURE 5:**
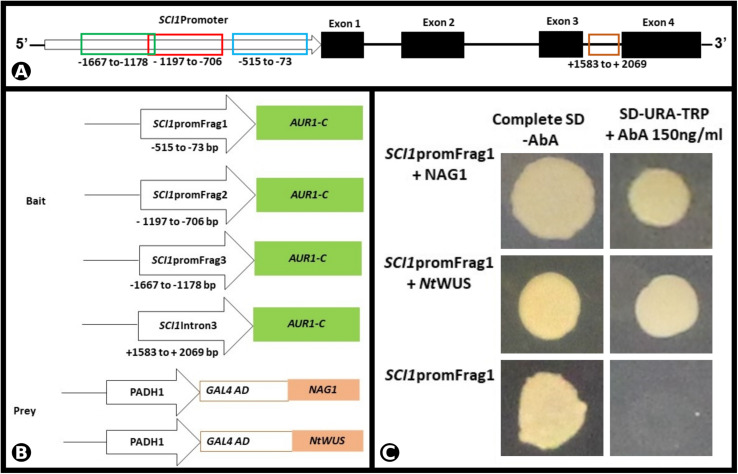
NAG1 and NtWUS associated with *SCI1* promoter in the Y1H assay. **(A)** Schematic representation of the *SCI1* genomic region, with the representation of exons and introns, and the fragments considered for the Y1H bait constructs. **(B)** Schematic representation of the bait and prey constructs used in the Y1H assay. The indicated promoter fragments of *SCI1* were used to make the *SCI1-AUR1-C* baits. **(C)** Physical interactions of NAG1 and NtWUS with fragment 1 of *SCI1* promoter in Y1H assays. Yeast expression plasmids pDEST22-NAG1 or pDEST22-NtWUS were introduced into yeast cells (PJ69-4a) carrying the reporter gene AUR1-C under the control of the different fragments of *SCI1* promoter. Eight independent transformants were cultured in synthetic defined media (SD/-Ura-Trp) in the presence of 150 ng/ml of Aureobasidin A (AbA). The empty vector pDEST22 was included as a negative control (last row).

To investigate if *SCI1* expression is regulated by the TFs NAG1 and NtWUS, we performed a Y1H experiment. Three different fragments of the *SCI1* promoter, as well as a fragment of the third intron (for details, see section “Materials and Methods”), were used as bait (Figures 5A,B). The Y1H results reveal that both NAG1 and NtWUS bind to *promSCI1Frag1* and allow the growth of colonies on the SD/-Ura/-Trp with 150 ng/ml Aureobasidin A (AbA) plates (Figure 5C) or 175 ng/ml AbA (data not shown). NAG1 and NtWUS do not bind to any of the other three genomic fragments tested in the Y1H assays (data not shown).

**FIGURE 6:**
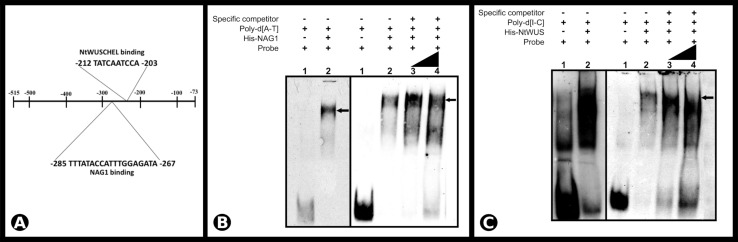
**(A)** Schematic representation of *SCI1* promoter (fragment 1) and the identified *cis*-acting elements for NAG1 (–285 to –267) and NtWUS (–212 to –203). **(B)** Electrophoretic mobility shift assay of NAG1 with the *SCI1* promoter sequence around position –285 to –267. Competitive EMSA using 77.5 fmols of the labeled probe; Lane 3 contains 5× unlabeled specific competitor; Lane 4 contains 10× unlabeled specific competitor. **(C)** Electrophoretic mobility shift assay of NtWUS with the *SCI1* promoter sequence around position –212 to –203. Competitive EMSA using 77.5 fmols of the labeled probe; Lane 3 contains 20× unlabeled specific competitor; Lane 4 contains 30× unlabeled specific competitor. Arrows indicate complexes between proteins and target sequences. The complete sequences of the EMSA probes are in Supplementary Figure 4. Poly-d(A-T) and Poly-d(I-C) are non-specific competitors.

Non-radioactive EMSA was used to demonstrate the binding of NAG1 and NtWUS with the respective putative binding sequences identified in the *SCI1* genomic promoter (for details of the double-strand oligonucleotides used, see Supplementary Figure 4). Both His-NAG1 and His-NtWUS, produced in *Escherichia coli*, caused mobility shifts when incubated with the corresponding ds-oligonucleotide (Figures 6B,C). Additionally, the EMSA analysis of the competition assay with increasing amounts of the unlabeled specific competitors exhibited a decrease of each DNA/TF complex and an increase of the free probe (Figures 6B,C). Our results demonstrate that NAG1 and NtWUS can bind to the sequences identified in the *SCI1* proximal promoter (Figure 6A) and may indicate that both NAG1 and NtWUS TFs regulate the *SCI1* gene expression.

**FIGURE 7:**
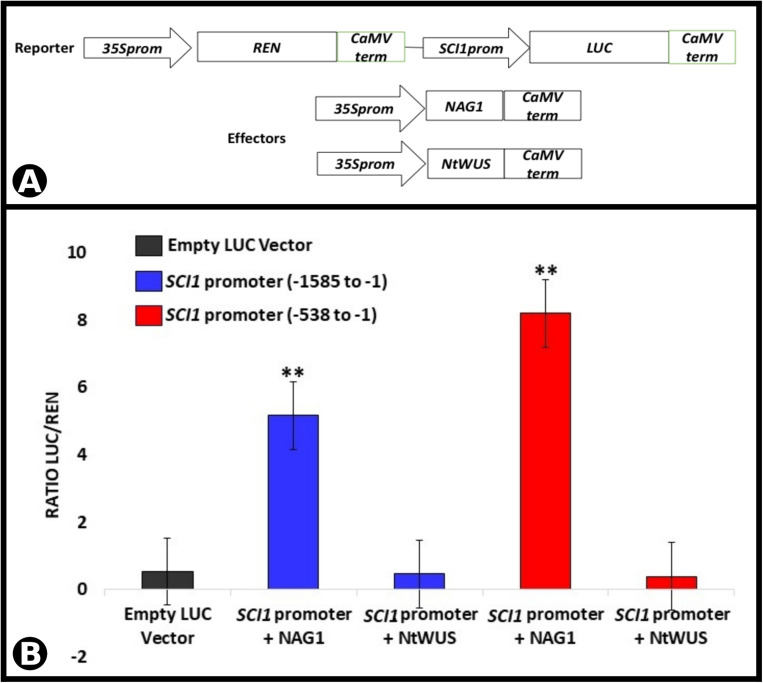
Luciferase activity assay performed by transient expression in *N. benthamiana* leaves driven by the *SCI1* promoter fragments. **(A)** Schematic representation of the reporter and effector constructs used in the luciferase activity assay. **(B)** Luciferase activity as a result of the interaction of NAG1 and NtWUS with *SCI1* promoter sequences indicated. Relative activity is the ratio LUC/REN. The expression of *REN* was used as an internal control. Data represent the means of three biological replicates. ^∗∗^Significantly different from the control (empty vector) by Student’s *t*-test (*p* < 0.01).

To confirm the binding of the TFs to the promoter sequence and assess their effects on *SCI1* expression, transient dual-luciferase assays were performed *in planta*. Two *SCI1* promoter fragments (for details, see section “Materials and Methods”), controlling the firefly luciferase expression (LUC), were tested for transactivation with NAG1 and NtWUS (Figure 7A). The results obtained for NAG1 had demonstrated a significant increase (*p* < 0.01) in luciferase activity in comparison with control (Figure 7B) when both fragments were used. On the other hand, NtWUS was not able to activate luciferase activity under the control of both *SCI1* promoter fragments (Figure 7B). As this assay was performed in leaf cells in which *SCI1* promoter is not expressed, only activation would be assessed. Then, it is still possible that NtWUS represses *SCI1* expression or depends on other TFs to induce it. Our results clearly show an activation effect of NAG1 in *SCI1* gene expression.

## Discussion

### Does WUSCHEL Regulate *SCI1* Expression at the Early Floral Meristem?

Our previous results have demonstrated that *SCI1* regulates cell proliferation in pistils of *N. tabacum* (DePaoli et al., 2011) and *A. thaliana* (DePaoli et al., 2014). Here, we demonstrated, through *in situ* hybridizations (Figures 1, 2) and analysis of *SCI1_*prom*_:SCI1-GFP* transgenic plants (Figure 3), that *SCI1* starts its expression in the young floral meristem and maintains it in the meristematic undifferentiated cells of all floral whorls. Additionally, we showed that *SCI1* is co-expressed with *NtWUS* in the floral meristem (Figure 4). The *WUS* expression in the *N. tabacum* floral meristem is broader than in the *A. thaliana* floral meristem. In *A. thaliana*, *WUS* expression is confined to the OC (Mayer et al., 1998; Adibi et al., 2016). However, WUS protein migrates from the OC, where it is found at the highest level, into adjacent cells via cell–cell movement and activates *CLV3* transcription (Yadav et al., 2011; Perales et al., 2016; Rodriguez et al., 2016). Therefore, the WUS protein acts more broadly, in a concentration-dependent manner, to spatially regulate transcription and maintain the homeostasis of the flower meristem (Perales et al., 2016). Despite the co-expression of *SCI1* and *NtWUS* in the floral meristem, *SCI1* expression is reduced in the OC (Figures 1G–I). So, we can speculate that high levels of WUS in the OC may suppress *SCI1* expression. It is known that WUS represses the expression of genes that can negatively influence the proliferation and indeterminate nature of meristematic cells (Ikeda et al., 2009). Examples of these genes are ARR5, ARR6, ARR7, and ARR15, which act in a negative feedback manner to regulate cytokinin signaling and inhibit cell proliferation in the meristem (Leibfried et al., 2005). Considering that SCI1 is a regulator/inhibitor of cell proliferation (DePaoli et al., 2011, 2014), its repression at the OC would be necessary to maintain the homeostasis of pluripotent cells.

As demonstrated by Y1H and EMSA (Figures 5C, 6C), *SCI1* is a direct target of NtWUS, and both genes are co-expressed in many cells of the young floral meristems. NtWUS may be responsible for the early activation of *SCI1* expression but cannot do it alone, as shown in the luciferase activity assay. Additionally, *WUS* is also expressed at the shoot apical meristem in different plant species (Laux et al., 1996; Mayer et al., 1998), whereas *SCI1* is only expressed in the floral meristem (several Figures shown here). Therefore, *SCI1* activation may be coordinated by NtWUS with some floral meristem identity gene, which is supported by the fact that putative *cis*-acting elements for *LFY* and *AP1* were found in the *SCI1* promoter. Future experiments will be necessary to determine the contribution of NtWUS is the regulation of *SCI1* expression.

### NAG1 Binds to the *SCI1* Promoter and Activates Its Expression

*Stigma/style cell-cycle inhibitor 1* and *NAG1* are co-expressed in cells of the floral meristem and young floral buds (Figure 4), suggesting *SCI1* as a possible target of NAG1. *In silico* analyses of the *SCI1* genomic sequence have identified several putative *cis*-acting regulatory elements, among which, AG binding sites. Putative AG binding sites were also found in the *SCI1* genomic sequence of the orthologs in *A. thaliana* (At1g79200), *Solanum lycopersicum* (Solyc05g008750.2), *Solanum tuberosum* (PGSC0003DMG400030526), *Oryza sativa* (LOC_Os02g07420), *Zea mays* (GRMZM2G010754), and *Glycine max* (08G16700). Most of the identified sequences correspond to non-canonical CArG-Boxes (Huang et al., 1993), but in *N. tabacum*, one of the identified regulatory domains represents a perfect CArG-Box (Aerts et al., 2018). Through Y1H, we demonstrated that NAG1 binds to fragment 1 (−515 to −73) of the *SCI1* promoter (Figure 5C), which contains a perfect CArG-Box (TTTATACCATTTGGAGATA). We also demonstrated that NAG1 binds specifically to the *SCI1* sequence (Supplementary Figure 4) containing the above mentioned CArG-Box by EMSA (Figure 6B). The luciferase activity assay showed that NAG1 interaction with the *SCI1* promoter significantly increased the reporter gene’s activity in relation to the control (Figure 7B). Taken together, these results demonstrate that the NAG1 TF binds to the regulatory region present in the *SCI1* promoter, activating its expression. Our data also reveal that NAG1 is necessary and sufficient to induce *SCI1* expression. Moreover, these results indicate that SCI1 acts downstream of NAG1, which, in addition to specifying the third and fourth floral whorls, is also responsible for the floral meristem termination (Mizukami and Ma, 1995; Wils and Kaufmann, 2017).

In Arabidopsis, AG is involved in regulating several genes related to flower development, among them *KNUCKLES*, *CRABS CLAW*, *JAGGED*, *NOZZLE/SPOROCYTELESS*, *SHATTERPROOF2*, *SEP3*, A*RABIDOPSIS THALIANA HOMEOBOX GENE1, AP1*, *AP3*, and even *AG* itself (Ó’Maoiléidigh et al., 2013). Gómez-Mena et al. (2005) reported that, at the beginning of floral organogenesis, AG regulates genes involved in the cell cycle, DNA repair, signal transduction, and maintenance of meristematic cells, among others. Additionally, a ChIP-seq experiment has identified almost 2000 AG targets, among which many transcriptional regulators and genes related to multiple cellular processes (Ó’Maoiléidigh et al., 2013). However, *SCI1* was not previously identified as an AG target, and this is novel information provided by our work.

In *N. tabacum*, after carpel specification, populations of meristematic cells are still maintained in different portions of the pistil, such as: in the fusion zone of the carpelar leaves, where subsequent style elongation and stigma development will occur; in placental primordium; placenta; and ovule primordia (Chang and Sun, 2020). As observed by *in situ* hybridization (Figures 1, 2) and *SCI1_*prom*_:SCI1-GFP* transgenic plants (Figure 3), *SCI1* is expressed in these tissues while cells proliferate, and its expression decreases when the female organ completes its development. Additionally, *SCI1* is expressed in the same pistil tissues in which *NAG1* is highly expressed (Figure 4). Mizukami and Ma (1995) have established that AG functions, in specifying the reproductive organs and determining the floral meristem, are separate and dependent on different levels of *AG* expression. They have demonstrated that the termination of the floral meristem requires high concentrations of AG. *AG* is a target of PERIANTHIA (PAN), which can increase *AG* expression (Wynn et al., 2014), and this direct regulation is involved in floral stem cell termination (Das et al., 2009). Additionally, the AG-dependent dose termination of the floral meristem was also observed in the double mutants of the PAN and SEUSS (SEU) genes (Wynn et al., 2014). Despite what is already known, there are still gaps in the downstream processes regulated by AG that culminate in floral meristem termination.

**FIGURE 8:**
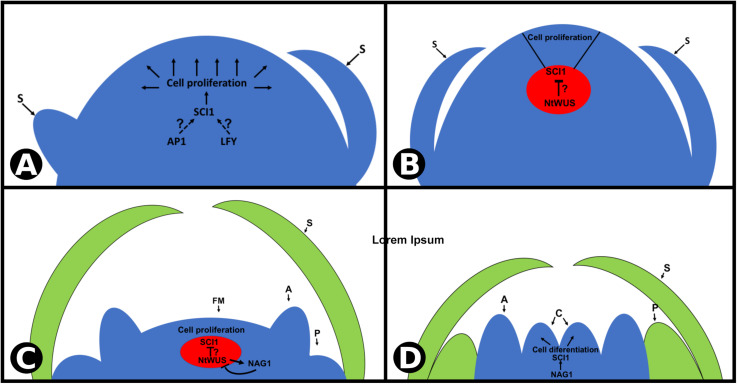
Proposed model for *SCI1* transcriptional regulation at the *N. tabacum* floral meristem. **(A,B)** *SCI1* expression is activated very early in development, as soon as the floral meristem is specified. It is expressed in all proliferative cells, except in the organizing center (in red), where a high NtWUS protein level is expected. *SCI1* activation at the floral meristem depends on TF(s) not yet identified (AP1 and/or LFY are possibilities according *in silico* analyses). **(C,D)** Later in flower development, *NAG1* is expressed at the two inner whorls (stamens and carpels) and drives the activation of *SCI1*. Then, *SCI1* is expressed in the same cells as *NAG1*, in all meristematic cells of the flower, until style/stigma and ovules differentiation. Floral meristem (FM), sepals (S), petals (P), anther (A), carpels (C).

Pistils are the last organs to be specified during flower development, and this occurs as a result of a feedback loop between WUS and AG (Lohmann et al., 2001). These genes act to maintain and terminate, respectively, the pluripotent cells in flower meristem. *SCI1*, as a target of both TFs, opens a way to understand the pluripotent cell homeostasis. A proposed model for *SCI1* activation and its role in regulating cell proliferation/differentiation is shown in Figure 8. As soon as floral meristem is specified, *SCI1* expression is induced by a TF(s) not yet identified, possibly LFY and/or AP1 (Figure 8A), based on the *in silico* analysis of *SCI1* promoter. It remains to be established which TF(s) is (are) responsible for the early activation of *SCI1* as soon as the floral meristem is specified (Figure 1).

At a later stage of floral meristem development, when sepals are being specified, *SCI1* expression is reduced on the OC (Figure 8B), which may allow the maintenance of the pluripotent cells. At stage −7, *SCI1* expression at the OC is still low, and NAG1 expression is activated. Based on what is known from Arabidopsis, we speculate that NtWUS may be responsible for repressing *SCI1* expression on the OC and for activating *NAG1* expression. Then, at a later stage, NAG1 may inactivate *NtWUS* in a negative feedback manner (Figure 8C). At stage −6, NAG1 takes over *SCI1* activation, and the fourth whorl is specified. Development progresses, and pistil cells divide to give rise to style, stigma, and ovules. At this point, pluripotent cells are no longer available, the last pistil tissues differentiate, and the flower meristem is terminated.

### Final Remarks

*Stigma/style cell-cycle inhibitor 1* is strictly expressed in floral meristematic cells, is activated by NAG1, and is expressed in the same pistil cells with high *NAG1* expression. Considering that SCI1 is a regulator of cell proliferation as demonstrated by the phenotypes of the *N. tabacum* transgenic plants (DePaoli et al., 2011) and the *A. thaliana* mutants (DePaoli et al., 2014), we suggest that SCI1 may act as an effector of the floral meristem termination, performing the fine-tuning of cell proliferation and differentiation. The fact that a SEP3 binding site was also found in *SCI1* promoter (Supplementary Table 1) and that tetramerization of AG and SEP3 is essential for floral meristem determinacy in Arabidopsis (Hugouvieux et al., 2018; Xu et al., 2019) strengthen our proposal. The identification of *SCI1*, a cell proliferation regulator, as a novel target of WUS and AG, contributes significantly to the understanding of flower meristem development.

## Materials and Methods

### Plant Material

*Nicotiana tabacum* cv Petit Havana SR-1 seeds were surface sterilized, germinated *in vitro*, and grown in Murashige and Skoog medium at controlled conditions (26°C temperature and photoperiod of 16 h light and 8 h dark). Leaf disks from *in vitro* grown plants were used for transformation via *Agrobacterium tumefaciens* with the gene construct *SCI1_*prom*_:SCI1-GFP*. Wild-type SR1 and transgenic plants were cultivated in a greenhouse in the city of Ribeirão Preto – SP, Brazil (latitude –21° 10′24″ S, longitude –47° 48′24″ W), with daily irrigations by automatic sprinkler, every 12 h.

### Microscopy Analyses

For bright field microscopy, the samples were fixed in 4% (w/v) paraformaldehyde and 4% (v/v) DMSO, dehydrated in a growing ethanol series, and included in base acrylic resin (Historesin Embedding Kit, Leica). Longitudinal sections of the floral axis, with a thickness of 2–3 μm, were obtained in a Leica^®^ RM2265 rotation microtome, with the aid of a high-profile, disposable steel razor (LEICA 818), and stained with 0.05% (w/v) toluidine blue, pH 4.4. The slides were mounted in Entellan mounting medium (Merck), and the images acquired under a microscope (Zeiss – Axiolab), equipped with a camera (Zeiss – Axiocam Color). For SEM, flowers from SR1 plants were dissected under a Wild M7A or Leica 165 FC stereoscopic microscope. They were fixed in 1% (w/v) glutaraldehyde and 4% (w/v) formaldehyde pH 7.2, submitted to vacuum (negative pressure of 760 mmHg) for 24 h, and kept in fixative solution at 4°C until use. They were then dehydrated in an increasing ethanol series, dried to a critical point with a CPD 030 (BAL-TEC) in CO_2_. Subsequently, the floral primordia were covered with a 15 nm gold layer in SCD 050 metallizer (BAL-TEC). The samples were analyzed under 10 kV voltage, and the electron micrographic record was performed using a scanning electron microscope JEOL 6060. A laser confocal microscope Leica TCS SP5 (Leica Microsystems Heidelberg, Germany) with the Leica LAS-AF Lite software was used for detecting fluorescence microscopy of GFP (argon laser; wavelength for excitation 488 nm and emission in the lengths between 500 and 590 nm). The LSM 7MP multiphoton microscope was used for excitation of thick samples, like floral buds (excitation using an 800 nm laser and detection in the spectrum between 500 and 540 nm). The autofluorescence of chloroplasts was detected in the range between 640 and 730 nm. Images were processed using the software Fiji-ImageJ 2.1.0/1.53c (Schindelin et al., 2012).

### *In situ* Hybridization

*In situ* hybridization was performed using digoxigenin-UTP-labeled probes, as described by Javelle et al. (2011). For the synthesis of the probes, the coding sequences (CDS) of *NAG1*, *NtWUS*, and *SCI1* were amplified by RT-PCR (primers are listed in Supplementary Table 2) and cloned in the TOPO^®^ TA Cloning^®^ Dual Promoter Kit vector. The probe synthesis was performed using the SP6/T7 Transcription Kit from Roche Life Science following the manufacturer’s specifications. The slides were photographed in a microscope (Zeiss – Axiolab) equipped with a camera (Zeiss – Axiocam Color).

### Yeast One-Hybrid Assay

The Y1H assay was performed using Clontech’s Matchmaker Gold Yeast One-Hybrid (Y1H) Library Screening kit. Three different fragments of the *SCI1* promoter and the third intron of the *SCI1* gene were amplified (primers are listed in Supplementary Table 2) and cloned into the pABAi-GW vector upstream of the AUR1-C coding sequence. The constructs pFrag1-abaiGW (−515 to −73bp upstream of the ATG), pFrag2-abaiGW (−1197 to −706 upstream ATG), pFrag3-abaiGW (−1667 to −1178 upstream ATG), pIntron3-abaiGW (+1583 to +2069 bp third intron) were sequenced and introduced in the PJ69-4A yeast genome by homologous recombination. The resultant yeast transformants were tested for self-activation of the AUR1-C gene and used in the Y1H assays. The CDS of NAG1 and NtWUS were cloned in fusion with the GAL4 activation domain at the pDEST22 vector (ProQuest^TM^ Two-Hybrid System with Gateway^TM^ Technology) and introduced in the yeast cells containing each of the genomic fragments mentioned above. The transformants were cultured in synthetic defined media (SD/-Ura-Trp) with or without 150 ng/ml of AbA for 3–5 days at 30°C.

### Production of Recombinant NAG1 and NtWUS Proteins and Electrophoretic Mobility Shift Assays

To produce the recombinant proteins NAG1 and NtWUS, each CDS was amplified (primers are listed in Supplementary Table 2) and cloned into the expression vector pDEST17 (Gateway^®^) in fusion with a HIS-tag. The CDS in the constructs were sequenced before use. These constructs were transformed into *E. coli* BL-21 (DE3) CodonPlus-RP. The production of 6xHis-NAG1 was induced with 0.5 mM IPTG for 8 h at 20°C, and of 6xHis-NtWUS was induced with 0.1 mM IPTG for 5 h at 30°C. In these conditions, it was possible to recover satisfactory levels of the desired proteins in the soluble fraction. For protein purification, the bacteria cells were lysed in 150 mM Tris–HCl pH 8 buffer, 150 mM NaCl, 10 mM imidazole, 1 mg/ml lysozyme, and cOmplete^TM^, Mini, EDTA-free Protease Inhibitor Cocktail (Roche; 11836153001) and sonicated four times using 20-s periods. Soluble proteins were purified using Ni Sepharose^®^ High Performance (Sigma; GE17-5268-01) and used for EMSA.

The double-strand probes (Supplementary Figure 4) used in EMSA were labeled with DIG-ddUTP using Roche^®^ Terminal transferase. The binding and electrophoresis assays were performed according to the DIG Gel Shift kit, 2nd Generation.

### Luciferase Assay

The *SCI1* promoter was amplified in two different fragments, a long one (−1585 to −1) and a small fragment close to the ATG (−538 to −1). Both fragments were inserted in the pGreenII 0800-LUC vector (Hellens et al., 2005), which has two different luciferase genes: the Renilla luciferase gene used as an internal control of the transient expression and the firefly luciferase gene under the control of the *SCI1* promoter sequences in this study (reporter construct). The *NAG1* and *NtWUS* CDS were cloned into the vector pK7WG2 (Karimi et al., 2002) for protein expression under the 35S promoter control and used as effectors. All gene constructs were sequenced before use. The reporter and effector constructs were introduced separately into *A. tumefaciens* strain GV3101. Different combinations of reporter and effector constructs were agroinfiltrated in *Nicotiana benthamiana* leaves for transient expression. The activity assay was performed 3 days after agroinfiltration. Firefly (LUC) and Renilla (REN) luciferases were detected with the dual luciferase assay reagents (Promega, Madison, WI, United States) using Costar^®^ 96-well plate. The promoter activity was calculated using the LUC/REN ratio. The data were analyzed by Student’s *t*-test.

## Data Availability Statement

Sequence data from this article is available at the National Center for Biotechnology site under the following accession numbers: SCI1 (LOC107802286), NtWUS (LOC107812471), and NAG1 (LOC107812878).

## Author Contributions

All authors have contributed to the intellectual content of this manuscript and have met the following three requirements: (a) significant contributions to the conception and design, acquisition of data, or analysis and interpretation of data; (b) drafting or revising the article for intellectual content; and (c) final approval of the published article.

## Conflict of Interest

The authors declare that the research was conducted in the absence of any commercial or financial relationships that could be construed as a potential conflict of interest.

## Footnotes

1 http://plantregmap.cbi.pku.edu.cn/binding_site_prediction.php

## Supplementary Material

Supplementary Figure 1 Negative controls of *in situ* hybridizations (*SCI1* sense probe).

Supplementary Figure 2 Anatomical and histological analyses of *Nicotiana tabacum* floral development (stage −2 to stage 1).

Supplementary Figure 3 Multiphoton microscope images of young flower buds of *SCI1prom:SCI1-GFP* transgenic *N. tabacum* plants.

Supplementary Figure 4 Sequences synthesized for EMSA with NAG1 and NtWUS.

Supplementary Table 1 List of *cis*-acting regulatory elements identified at *SCI1* genomic sequence by PlantRegMap.

Supplementary Table 2 Primers used to amplify and sequence the different DNA fragments and CDSs used in this work.
