# Supplementary material for: SCI1 Is a Direct Target of AGAMOUS and WUSCHEL and Is Specifically Expressed in the Floral Meristematic Cells
Source: Front Plant Sci. 2021 Mar 18;12:642879. doi: 10.3389/fpls.2021.642879 (PMC8012853; doi:10.3389/fpls.2021.642879)
Supplement: Supplementary Figure 1 — Negative controls of in situ hybridizations (SCI1 sense probe). [file Data_Sheet_1.docx]

**Supplementary Figures**

**
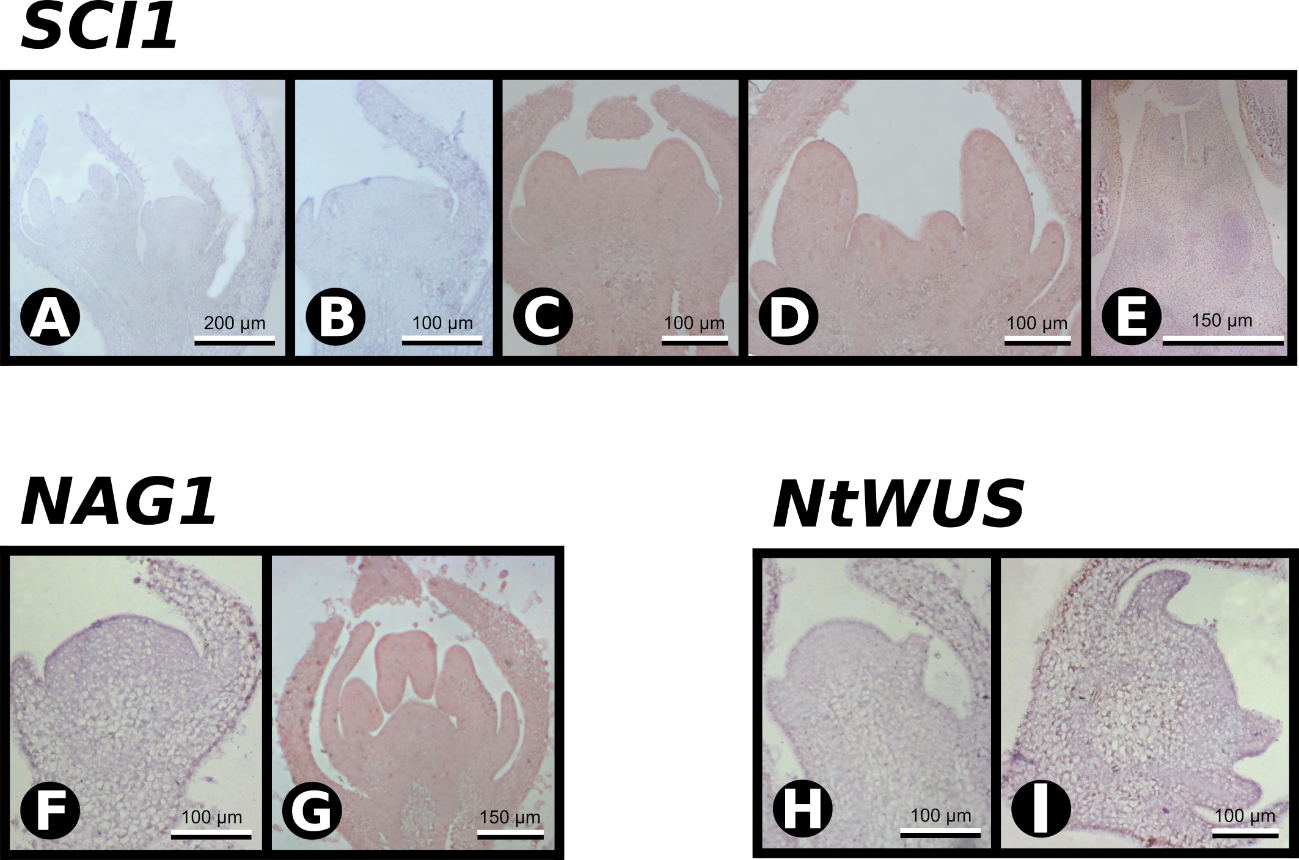
**

**Supplementary Figure S1.** *In situ* hybridization of flower buds in different developmental stages with *SCI1* sense probe (negative control).

Negative controls of in situ hybridizations (SCI1 sense probe).

**
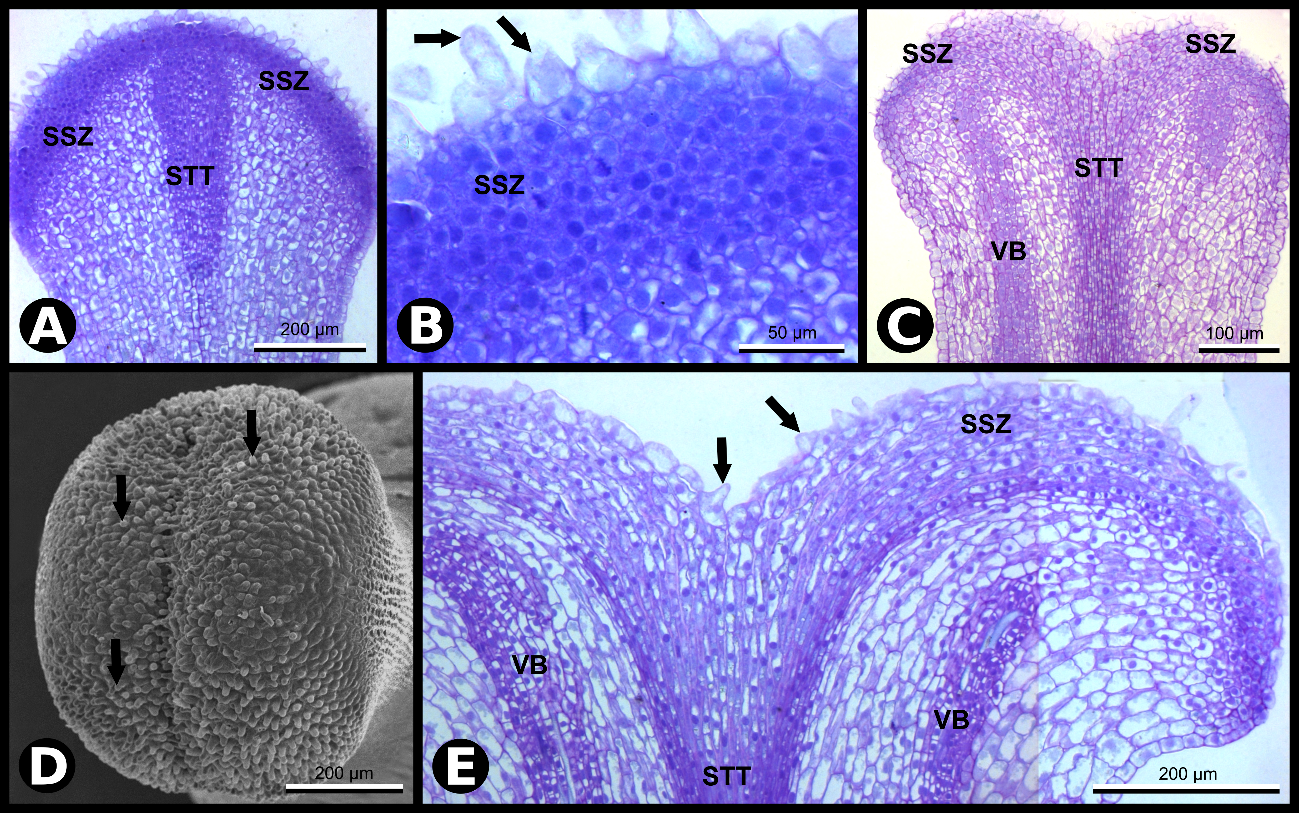
**

**Supplementary Figure S2.** Anatomical and histological analyses of *Nicotiana tabacum* floral development. (A) Bright-field microscopy of a longitudinal section of the stigmatic region at stage -2. Scale bar: 200 µm. (B) A higher magnification view of the stigmatic secretory zone in A, showing the papillar cells (arrows). Scale bar: 50 µm. (C) Bright-field microscopy of a longitudinal section of the stigma/style at stage -1. Scale bar: 100 µm. (D) Scanning electron microscopy (SEM) of the stigma at developmental stage -1/1. The stigma is already established and covered with papillar cells (arrows). Scale bar: 200 µm. (E) Bright-field microscopy of a longitudinal section of the stigmatic region at stage 1. The specialized tissues of the stigma/style, stigmatic secretory zone (SSZ) and the beginning of the stylar transmitting tissue (STT) are evident. Arrows show the papillar cells in the stigma surface. Scale bar: 200µm. Vascular bundles (VB).

**
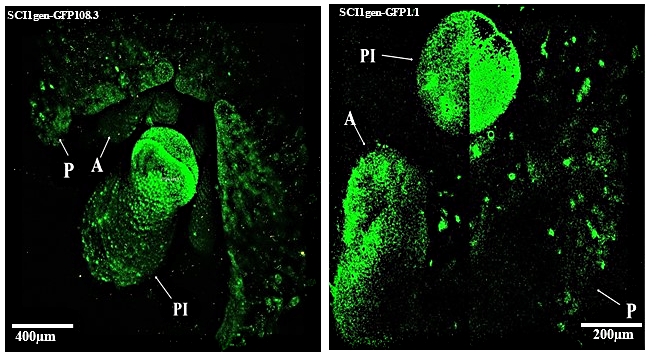
**

**A**

**Supplementary Figure S3.** Multiphoton microscope images of young flower buds of *N. tabacum*. (A) Flower at developmental stage -3 (Koltunow et al., 1990) from SCI1gen-GFP108.3 transgenic plant. Scale bar: 400µm (B) Flower at developmental stage -2 (Koltunow et al., 1990) from SCI1gen-GFP1.1 transgenic plant. Scale bar: 200µm.

In both images, the three floral whorls, highlighted by the arrows, express SCI1-GFP protein. Petals (P), anthers (A), pistil (PI).


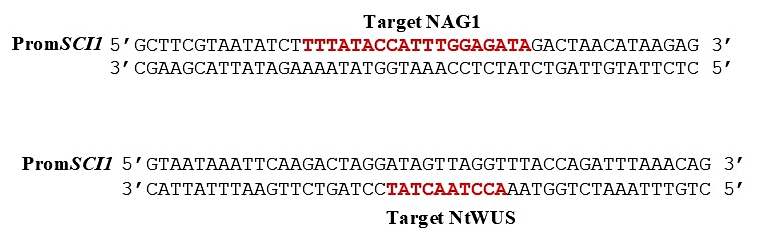


**A**

**B**

**Supplementary Figure S4.**

(A) Sequence synthesized for electrophoretic mobility shift assay (EMSA) with NAG1. (B) Sequence synthesized for electrophoretic mobility shift assay (EMSA) with NtWUS.
